# Medical and Philosophical Intelligence

**Published:** 1811-04

**Authors:** 


### MEDICAL AND PHILOSOPHICAL INTELLIGENCE.

ROYAL SOCIETY.—Jan. 31. A paper by Mr. Home was read on the nonconducting powers of the thoracic duct and the spleen from the stomach to the bladder. Mr. H. related a number of experiments made with ligatures, or by removing the spleen and keeping a ligature on the thoracic duct, in which state rhubarb was taken into the stomach of the animal, and was detected in the urine; whence he inferred, contrary to some opinions he formerly published, that those two organs are not necessary to the secretion of urine.

Feb. 7. Mr. Smithson's paper on zeolite was read. This ingenious mineralogist having received some specimens of this mineral from M. Haüy himself, and labelled by his own hand, he deemed it a favourable opportunity of ascertaining if there were any chemical difference between the mesotype of the French crystallographer, and zeolite or the natrolith of Klaproth, as he had previously discovered the existence of soda in all the specimens of zeolite which are found in these kingdoms, as well as those in Germany. M. Vauquelin analysed several specimens of zeolite without discovering any traces of soda; but Mr. Smithson discovered alkali even in the mesotype sent him by M. Haüy, and in every other specimen of zeolite in his possession. From this circumstance he is inclined to prefer the original name of zeolite, as given to this mineral by its discoverer Cronsted, in preference to that of mesotype given it by Haüy; and considers the distinction between mesotype and natrolith as unsupported by chemical analysis.

Feb. 14. A long paper on the effects of vegetable poisons on animals by Mr. Brodie was read. The author has pursued his researches for a considerable time, and detailed to the society the result of his experiments on rabbits, cats, and dogs, with alcohol, oil of bitter almonds, extract of aconite, tobacco, &c. These vegetable substances thrown into the stomachs of dogs, cats, rabbits, &c. instantly killed them by acting on the nervous system, and producing a compression of the brain: thrown into the rectum, the same effects were produced. The pulsation and heat of the heart, after administering these poisons, were maintained for a considerable time by means of artificial respiration, except with tobacco, which instantly destroyed the powers of the heart, and arrested the pulsations. One drop of the empyreumatic oil of tobacco let fall on the tongue of a cat killed her, but did not destroy the pulsation so instantaneously. Mr. Brodie made a great number of experiments with the vegetable poison used by the American Indians to poison their arrows, and with nearly similar results.

On Thursday, the 21st of February, a paper was read by H. Davy, Esq. LL.D. Sec. R.S. on a gaseous combination of oxymuriatic gas and oxygen.

Mr. Davy procured this extraordinary body by acting on hyper-oxymuriate of potash by diluted muriatic acid. Mr. Davy stated, that it explodes by the application of a heat equal to that of the human body; and that though the oxygen and oxymuriatic gas expand in separating from each other, yet heat and light are produced. The metals which burn readily in oxymuriatic gas do not act upon this body till it is decomposed. He described a number of properties of this compound, all of which he considered as strengthening his opinion of the undecompounded nature of oxymuriatic gas; and as particularly opposed to the idea of its containing oxygen. Mr. Davy proposed the name zuthine or zuthic gas for this body from its colour, which is bright yellow; but he stated that he should be content to adopt any other name which might be considered as more appropriate. *Phil. Mag.*

---

Royal society of edinburgh.—On the 7th of January, Sir George Mackenzie continued his account of the mineralogy of Iceland, and described some very curious geological facts. On the 21st he concluded his mineralogical detail, with an interesting description of Mount Hecla, and other volcanic districts. In this paper Sir George made some remarks which tended to place obsidian and pumice in a conspicuous point of view, as relating to the different theories of the earth, and clearly proved their origin to be igneous; a position which has hitherto been denied by Werner and his pupils.

On the 4th of February, Dr. Brewster read an ingenious paper on the longitude of the comet of 1770. Sir George Mackenzie described some remarkable hot springs in Iceland. To one of these he gave the name of the *alternating Geyser*, as it spouted from two distinct orifices evidently connected within, but only from one at a time, whose operations alternated with those of the other intervals of time.

On the 18th, Professor Playfair read part of a biographical sketch of the late John Robison, LL. D. and Professor of Natural Philosophy in the University of Edinburgh.—Mr. Allan communicated a letter from Dr. Henry, of Manchester, describing the position of some singular masses of a substance apparently composed of wax and rosin, which had been laid bare by a late overflow of the river Mersey, a little below Stockport, about three feet under the soil,—and supposed to be the refuse of some manufactory, of which no other vestige or recollection now remains. *Phil. Mag.*

---

French national institute.—Abstract of the Report of the National Institute at Paris for the year 1810.

Messrs. Gay-Lussac and Thenard have directed their attention to compare the relative powers and energies of different Galvanic piles. They have discovered that the force of the pile is not increased in proportion to the number of plates. To produce a double effect, the number of plates must be increased eight times. In general, it was found that the quantity of gas the piles will produce, duce, is nearly in proportion to the cube root of the number of plates employed.—Amongst the discoveries to which the Galvanic pile has given rise, there are few more interesting to general chemistry than the transformation of the alkalies into combustible substances of metallic splendour.

This transformation, first discovered by Mr. Davy, was afterwards doubted by Messrs. Lussac and Thenard. In their former report they were disposed to consider potassium and sodium as combinations of the alkalies with hydrogen, and to class them amongst the compound substances called hydrurets: subsequent experiments have led them to incline to the opinion of Mr. Davy, and to regard potassium and sodium as simple metallic substances.

M. Berthollet has communicated a process for making the muriate of mercury, called mercurius dulcis or calomel, by passing oxygenated muriatic gas through mercury; it combines rapidly with the metal, and forms with it the muriate of mercury; and as this metallic salt has a perfect analogy with other mercurial salts produced by other acids, and mercury at the minimum of oxidation, he concludes that the mercury, in forming this combination, has been reduced to an oxide by the oxygen of the acid, and not by that of the water.

M. Guyton has directed his attention to the mode of giving a permanent red colour to glass, by means of copper, which by accident he first discovered might be done. M. Sage has also taken a part in these experiments, with the intent to colour glass red by means of copper and the phosphate of lime, or with bones; and he has shown crystals of glass, from the bottom of the pots used to melt glass in the bottle-manufacture at Seves, which had some resemblance to hexaëdral prisms. It is well known that simple means have been discovered to extract soda from common salt. France formerly imported this article, so necessary to the arts: an inconvenience attended the mode of preparing it, from the quantity of acid gas which escaped, and was highly injurious. Amongst the different means of preventing this inconvenience which have been attempted, that of M. Pelletan the younger is deserving of notice. It consists in making the muriatic gas pass through long horizontal tubes partly filled with calcareous earth, which absorbs the gas, forming with it the muriate of lime. The experiments of M. Sage on plumbago (black lead) show that this substance does not contain any iron, but consists of a coaly matter mixed with one-tenth part of clay. The fossil carbon of St. Symphorien, near Lyons, approaches nearer to this substance than any other known mineral.

M. Deyeux has presented to the class of agriculture a loaf of sugar made from the red beet (*betterave*), which had all the whiteness and flavour of the sugar from the cane. He has announced that this substance may be made in great quantities by the proprietors, who have devoted to this attempt 400 acres of ground. Should it succeed on the great scale, it will change the relations of the two worlds.

Medical Society of London.—This Society held its Anniversary Meeting on the 8th of March.

The customary Oration was given by *Mr. Blair;* its subject, the *Origin,* nature, progress, and diffusion of Syphilis.

Mr. Blair assumed that *lues venerea* was not brought from America to Europe—that it was not known to the ancient medical writers, or noticed by any one before 1493—and, therefore, that it was generated in Europe about that period.

We apprehend this to be a correct statement of the data on which the oration was founded; and we have no fear of being contradicted when we assert, that the orator manifested great powers of research, a respectable portion of erudition, and considerable ingenuity in the employment of his materials.

Mr. Blair's opinion of the non-existence of *lues venerea* in the new World, was founded on a close examination of the early Spanish historians, who had been quoted by Friend, Astruc, and Gintanner, to prove the American origin; so that Mr. Blair did not adopt his opinion, in opposition to them, without first weighing the whole evidence. The total silence of medical authors respecting this disease, prior to the end of the 15th century, was his chief motive for concluding that Syphilis was unknown to remoter ages; and although Hensler, Sanchez, Gruner, Sprengell, &c. &c. have attempted to throw light on the appearance of this disease in Europe, Mr. Blair did not acquiesce in the sentiments of any of those learned authors. He likewise combated the ideas of several English writers and others, to whom we owe certain hypotheses on the generation and first production of *lues venerea;* especially the notions of Sydenham, Harvey, Lettsom, and Howard,—who suppose the disease to have arisen from a combination or modification of two other affections, or from the conversion of leprosy or yaws into Syphilis. These suppositions were rejected on historical grounds, or for want of sufficient evidence.

Toward the conclusion of this spirited, and what the painters call finished sketch, Mr. Blair dwelled particularly upon a fortuitous concurrence of incidents, as remarkable, as they were important in the affairs of men. The discovery of America, the wars that desolated the fairest provinces of Europe, and a pestilence which spread with wasting destruction over Spain, Germany, Italy, and France, marked the close of the 15th century. It appeared to us, that Mr. Blair looked to this awful and dire epidemic, perhaps, in conjunction with the aggravation of all human calamity by war, for the *origin of Syphilis.*

The source of this disease has been sought by philosophers, historians, and physicians, with little success; and if the importation of *lues* from America be denied, its origin is yet left in utter darkness. Though the opinion of Mr. Blair may not be original, we expect he will illuminate the obscurity in which the question of the origin of Syphilis has hitherto remained; and therefore we hope he will accede to the Request of the Society, and publish his Oration; with a fair statement of his evidence, and a full reference to his authorities.

*Officers for the ensuing Year.*

President . . . . George Pinckard, M. D.

Vice-

| | |
|---|---|
| Vice Presidents . . | James Hamilton, M. D.<br>Richard Temple, M. D.<br>Ludford Harvey, Esq.<br>John Abernethy, Esq. |
| Librarian . . . . | H. Clutterbuck, M. D. |
| Secretaries . . | George Birkbeck, M. D<br>John Mason Gord, Esq. |
| Secretary for Foreign Correspondence | Charles Taylor, M. D. |
| Treasurer . . . | Sayer Walker, M. D. |
| Registrar . . . | A B. Turnbull, Esq. |

---

### *Hospitals for the Small-Pox and Inoculation, St. Pancras.*

ANNIVERSARY, THURSDAY, the 14th March, 1811.

The House Committee avail themselves of the present return of their Annual Meeting, for recalling the public attention to the great Utility of this Establishment, exemplified by its beneficial effects during the past year.

They desire to remind a liberal Public of the importance of an Institution devoted to the relief of persons under circumstances peculiarly distressing to themselves—often dangerous—and always inconvenient to their relatives and others:—an Institution by which the treatment of a most malignant disease has been much improved, and the means of its communication in a great degree lessened—an Institution, to the close investigation and faithful reports of which *Vaccination* is in a great degree indebted for its general reception with the Public.

Into the House appropriated for the reception of poor patients afflicted with the casual Small-Pox, 149 have been received:—some of them unfortunately became victims to the extreme malignity of their cases; but by far the greater part have been restored to their respective families and occupations. Into the House for Inoculation, 127 Patients have been admitted, all of whom have recovered:—and, in order to secure Infants thereby, (even before they can be removed from their mothers), thirteen of the latter have, at their own desire, been admitted, with their offspring, on payment for their own board.

In addition to these, the benefit of Vaccination has been given to 1,720 Out-patients.

Thus have these Hospitals afforded relief to One thousand Nine Hundred and Ninety-six poor persons in the year 1810; and from the period of their first establishment in the year 1746, to the present time, they have protected and relieved no less than 96,329 poor of this metropolis and its neighbourhood, without fee or expense.

Hence it is obvious that this Institution has greatly alleviated the affliction of the casual Small-Pox; that it has, with scrupulous observance, met the recent orders of the Governors, in limiting the Inoculation of the Small-Pox within its own walls—while it has given, and is daily giving, with alacrity, the benefits of Vaccination to all who apply for for it; thus fulfilling the original Plan of the Establishment, happily expanded by the early adoption of that Discovery which, under the blessing of Divine Providence, may be hailed as one of the mercies vouchsafed to the present race, and as the harbinger of that more fortunate period when the dread of casual Small-Pox shall cease by the universal adoption of Vaccination.

Until that wished-for period, which every humane mind hails with cheering anticipation, when the object of the Governors, in common with the benevolent part of the public, shall be so far accomplished, it becomes almost superfluous to urge the obvious necessity of a Small-Pox Hospital, as a place of refuge for Patients seized with the casual Small-Pox; and for preventing that direful disease from spreading by contagion.

Those who see most of the Poor will be sensible of their want of prudence in matters of the highest importance to their health and lives. The industrious mechanic, whilst anxiously procuring daily support for his children, is too often inattentive to repeated admonitions, and even frightful examples, which should teach him the hourly danger to which they are exposed; at the age when their industry is most required, they arrive at the metropolis, obnoxious to this dreadful malady, and too often are irrecoverably lost, before they or their friends are aware of their danger. The hardy peasant is in still greater danger, in proportion to the strength of his frame; but most of all the Defenders of our Country, and others who, after breathing the pure air of the ocean, arrive at the infected lodging-houses on the banks of the Thames, or in the neighbourhood of the Docks; many of such persons might have been entirely lost, had not this Institution received and rescued them from imminent danger.

It is with heartfelt satisfaction that the Committee can assure the Public, that the mortality from Small-Pox has been evidently less, during the ten years which have elapsed since the introduction of Vaccination, than in any equal number of years, in succession, for a preceding century; and comparing the one with the other, and reflecting that the victims to the Small-Pox are chiefly, if not entirely among the Poor, the Committee are fearful that many lives are lost for want of timely application to this Hospital, through ignorance of its establishment, or other causes. And although the annual income arising from its funds is inadequate to its present expenditure, yet neither are their exertions lessened nor their industry in the discharge of their duty relaxed. They therefore scruple not to request the diligent co-operation of every well-disposed individual, in giving publicity and encouragement to this Institution—to the necessity of its continuance—and consequently to its support.

A Donation of 31l. 10s. constitutes a Governor for Life; and 5l. 5s. an annual Governor: and any other Benefactions, or Legacies, are gratefully acknowledged.

By the Committee, 6th February, 1811.

A. HIGHMORE, Secretary,
Solicitor, 33, Ely-Place.

**SMALL POX AND INOCULATION HOSPITALS, PANCRAS.**—*Report given at the Anniversary Meeting of the Governors of this Charity, the 14th day of March,* 1811.

| | Inoculated | | Vaccinated | | | |
|---|---|---|---|---|---|---|
| | In Patients | Out Patients | In Patients | Out Patients | Total | Of whom died |
| From Jan. 21, 1799, to Dec. 31, 1801. | — | — | 1580 | 5912 | 7492 | |
| During 1802 | 49 | 239 | 337 | 3790 | 4415 | none |
| 1803 | 33 | 230 | 144 | 2658 | 3065 | none |
| 180S | 67 | 2'1 | 89 | 2551 | 2988 | none |
| 1805 | 300 | 2338 | 50 | 2016 | 4784 | Two Inoculated Out Patients. |
| 1806 | 320 | 2245 | 22 | 945 | 3532 | Two ditto Out Patients |
| 1807 | 348 | 4246 | 44 | 1577 | 6215 | Five ditto Out Patients. |
| 1808 | — | — | — | — | | One ditto Out Patient, and |
| to 5th May | — | — | 6 | — | 2548 | also one ditto by Dropsy |
| to 31st Dec. | 264 | 1032 | — | 1246 | | in the Head. |
| 1809 | 136 | — | — | 1515 | 1651 | none |
| 1810 | 127 | — | — | 1720 | 1847 | none |
| | 1644 | 10.611 | 2272 | 23.960 | 38.487 | |

The Register of these Hospitals shews, that out of one thousand six hundred and forty-four In-Patients inoculated during nine years, terminating the 31st of December last, none had died; and that out of ten thousand six hundred and eleven Out-Patients inoculated between the 1st of January, 1802, and the 5th of May, 1808, (when the practice was discontinued by order of the Governors) ten deaths have been ascertained; whilst the mortality of those who receive the disease casually is calculated in the proportion of one in six.

Dr. Clough, Physician, and Man-midwife to the St. Mary-le-bone General Dispensary, &c. will, on Monday morning, the 8th of April, at half-past ten, commence his Spring Course of Lectures on the Theory and Practice of Midwifery, including the Diseases of Women and Infants, as connected with it, at his Lecture Room, 68, Berners Street, Oxford Street. The Evening Lectures are continued as usual, Tuesdays, Thursdays, and Saturdays, at Seven.

Dr. Clough embraces this opportunity of expressing his high gratification to those numerous Students who have honored his school, for their regular and uniform attention; at the same time to announce his intention of establishing, shortly, an Obstetrical Converzatione every fortnight during the Winter, to which they only can be admitted. And by way of emulation and forwarding their views, he purposes presenting annually, toward the conclusion of the season for anatomical pursuits, to the authors of the best paper, or dissertation on a subject relative to the science, a SILVER MEDAL for which also they only can be candidates. Rules and regulations, Prospectus, and Syllabus of the Course may be had as above, and of the Medical Booksellers.

---

Dr. REID's next Course of Lectures on the Theory and Practice of Medicine, will commence on Wednesday, the 1st of May, at nine o'clock in the morning, at his house, Grenville-street, Brunswick-square.

---

Mr. SINGER will commence his Lectures on the Chemical Agencies of Electricity, on Thursday, the 11th of April. The Voltaic Apparatus constructed for the Illustration of these Lectures, exposes upwards of 14,000 square inches of zinc surface.

---

Dr. Hutton's Dictionary of Mathematics and Philosophy is ready for the press, and may be expected to be delivered to the public with all his own improvements, made from the late discoveries in those sciences, for the collecting of which he has assiduously employed himself ever since the time of the first publication of that valuable work.

---

The first volume of the Transactions of the Geological Society, in 4to. with many plates, is in the press, and will be ready for publication in the month of May next.

---

Mr. Parkinson has announced the publication of the third and concluding volume of Organic Remains of a former World. Containing the fossil remains of echine, shells, insects, crustaceæs, fishes, amphibiæ, quadrupeds, &c.; with twenty-three coloured plates. It will be published in the middle of June.

---

The London Dispensatory, on which Mr. Anthony Todd Thomson has been for some time at work, is now almost ready for publication.

cation. It will contain the botanical descriptions of the vegetable substances of the Materia Medica; and several other important improvements.

